# THE RELATIONSHIP BETWEEN EMPLOYEE MOTIVATION AND PROFESSIONAL BURNOUT AMONG NURSES IN GERMANY: AN ANALYTICAL STUDY

**DOI:** 10.13075/ijomeh.1896.02638

**Published:** 2025

**Authors:** Katarzyna Swakowska, Dominik Olejniczak, Łukasz A. Poniatowski, Anna Staniszewska

**Affiliations:** 1 Gute-Zeit-Pflege24 APD GmbH, Obernburg am Main, Germany; 2 Medical University of Warsaw, Department of Public Health, Warsaw, Poland; 3 Dietrich-Bonhoeffer-Klinikum, Department of Neurosurgery, Neubrandenburg, Germany; 4 Medical University of Warsaw, Department of Experimental and Clinical Pharmacology, Warsaw, Poland

**Keywords:** nurses, burnout syndrome, *Maslach Burnout Inventory*, Germany, *Areas of Worklife Survey*, *Work Extrinsic and Intrinsic Motivation Scale*

## Abstract

**Objectives:**

The purpose of the following paper is to elaborate on the connections between motivation and professional burnout. The analytical study will concern nurses employed in Germany. In particular, the study focuses on explaining how different types of motivation influence burnout indicators.

**Material and Methods:**

The study employed a quantitative research approach using the *Areas of Worklife Survey* (AWS), the *Work Extrinsic and Intrinsic Motivation Scale* (WEIMS), and the *Maslach Burnout Inventory* (MBI) to assess burnout and motivation among 301 nurses.

**Results:**

The results revealed that nurses with higher intrinsic motivation experienced lower burnout levels, while those relying on external regulation and amotivation exhibited greater emotional exhaustion and depersonalization. Moreover, older and more experienced nurses reported higher burnout levels and declining intrinsic motivation, while younger nurses demonstrated stronger engagement but faced significant workplace stress. Workplace factors such as fairness, workload, and rewards were found to significantly influence motivation and burnout.

**Conclusions:**

The findings emphasize the need for healthcare institutions to implement strategies that would support development of intrinsic motivation and improve working conditions to reduce burnout and support long-term professional engagement of nurses.

## Highlights

- Higher intrinsic motivation correlates with lower burnout.
- The longer the time in the nursing profession, the higher the level of burnout.
- Salary has little impact on the level of burnout.

## INTRODUCTION

In the contemporary work environment, the phenomena such as job satisfaction, motivation and burnout have a significant influence over the general condition and performance of employees [1,2]. The available literature indicates that those problems are highly complex and stresses the importance of strategies designed to reduce burnout while enhancing employees' motivation.

The most distinguishing features of professional burnout include emotional exhaustion, depersonalization and a reduced sense of personal achievement, which in general are recognized as a major obstacle to job satisfaction and workplace morale [3]. Maslach and Leiter [4] highlight that burnout is particularly common in professions centred on human services, where frequent interactions with people contribute to emotional fatigue and decreased effectiveness. These findings correspond with research by Richemond et al. [5], who argue that burnout depletes the energy necessary for efficient task performance and fosters frustration and tension stemming from perceived failure.

Among nurses, burnout is linked to lower turnover rates due to exhaustion and declining motivation [6]. In addition to that, low job satisfaction worsens burnout, as nurses frequently encounter numerous stressors. Interest in burnout grew significantly in the 1970's, with increasing scholarly attention to the issue [6]. In response, healthcare managers organized dedicated sessions to address its detrimental impact on workplace productivity [5].

The nursing profession has become increasingly demanding and stressful due to various risk factors, workplace conditions and the diverse challenges of this profession [7]. Nurses are required to deliver patient care with empathy while maintaining high levels of concentration in a pressurized environment. According to Dimunova et al. [8], key factors that contribute to burnout among representatives of this specific occupation include excessive workloads, time pressures, shift work and the specific demands of different departments. Additional risks are rooted in organizational culture, particularly workplace relationships, conflict management and insufficient resources.

Farivar and Esmaeelinez [9] and Trépanier et al. [10] had provide important insights into the relationship between motivation in the context of occupational burnout. In particular, Farivar and Esmaeelinez had examine how various forms of motivation, combined with personality traits such as extraversion, has an impact over the bond that exists between job demands and burnout [9]. The study that was mentioned above elaborates on the influence of motivational factors in controlling and eventually – minimizing burnout. In a similar way Trépanier et al. come to the conclusion that the quality of work motivation can have a moderating influence on job resources and burnout. Their findings suggest that motivation not only shapes employees' perceptions of job demands but also affects how they utilize resources to manage burnout effectively [10].

Expanding on the concept of balancing job demands and resources, Bebiroglu et al. [11] explore how this equilibrium can serve as a predictor of job burnout. Their study highlights the necessity of ensuring that workplace demands are effectively aligned with available resources, as they emphasize this balance as a key factor in preventing burnout. This perspective provides valuable insights into how organizational structures and workplace policies can be shaped to reduce burnout and promote employee well-being.

Furthermore, Tamaela et al. [12] examine the beneficial role of intrinsic motivation in lowering burnout and increasing job satisfaction. Their research reinforces the idea that cultivating intrinsic motivation among employees can be an effective approach to reducing burnout while enhancing overall job satisfaction.

The objectives of this article are:

– to analyze the relationship between motivation and professional burnout among nurses employed in Germany,
– to determine how different types of motivation correlate with burnout indicators,
– to assess the impact of demographic factors on motivation and burnout levels.

## MATERIAL AND METHODS

In this study the authors used 3 questionnaires. First the *Areas of Worklife Survey* (AWS) by Leiter and Maslach [13] was used. This particular research tool attempted at evaluating the scope of perceived burnout among the participants of the study. The AWS specifies 6 areas that are particularly relevant in this regard: workload, control, reward, community, fairness and values. In the questionnaire, the research sample was encouraged to base their opinions upon the Likert scale, where 1 – strongly disagree, 2 – rather disagree, 3 – difficult to say, 4 – rather agree, 5 – strongly agree. The score for each scale was calculated as the mean of the individual statement ratings.

The second research instrument that was used in this study was *Work Extrinsic and Intrinsic Motivation Scale* (WEIMS). This particular tool consists of 18 items that were designed by Tremblay et al. [14] in order to measure work motivation. The author of this study used the Polish version of this questionnaire (WEIMS-PL) which includes 6 subscales that include: amotivation (AMO), external regulation (EXT), introjected regulation (INTRO), identified regulation (IDEN), integrated regulation (INTEG) and intrinsic motivation (IM). Again, the answers of the respondents were placed upon a Likert scale (7 pts in this case).

The third research instrument used in this study was *Maslach Burnout Inventory* (MBI). It was designed in order to measure burnout. The tool concerns the aspects of this phenomenon such as emotional exhaustion, depersonalization as well as decreased professional efficacy [15]. In the questionnaire, 22 statements were included and the participants of the study were asked to place their answers with 4-point scale: 0 – never, 1 – rarely, 2 – often, 3 – very often. Scale's items include: emotional exhaustion, depersonalization, personal accomplishment.

For the purposes of the study, the Polish versions of all scales were utilized.

The significance of differences in score distributions between the groups was evaluated using the Mann-Whitney U test. Associations among the variables were evaluated by Spearman's rank correlation coefficient. Non-parametric tests were chosen because the variables did not meet the assumption of normal distribution. The normality of the analyzed variables was tested using the Shapiro-Wilk test. A value of p < 0.05 was considered statistically significant. Statistical analysis was performed using Statistica 13 (StatSoft Inc., Tulsa, USA).

The study was conducted in the period of January 2024 – March 2025. A cross-sectional study of 394 nurses working in Germany, in palliative care, hospice, primary healthcare, emergency medical, or hospitals was conducted. Before commencement of the study, nurses were informed about the principles of anonymity and confidentiality in data collection. The completion of the survey meant that the nurses gave their consent to participate in this study. The study was approved by the Ethics Committee of the Medical University of Warsaw, Poland (No. AKBE/99/2025), as this study is a part of the larger study conducted in Poland and Germany and supervised by Medical University of Warsaw.

## RESULTS

There were 301 totally filled out questionnaires (response rate 76%), including 89% woman and 11% men. The age of the respondents was mean (M) ± standard deviation (SD) 39.5±12.94 years old. The work experience of the respondents was M±SD 11.8±10.33 years. The net monthly salary of the respondents was M±SD 2735.2±302.30 EUR.

All of the participants of the study had at least a secondary education. The highest number of participants were employed in the surgery (16%), paediatrics (16%), internal medicine (13%), orthopaedics (9%), primary healthcare (7%), nephrology (7%), emergency (6%) and neurology (4%) departments. Other departments were represented by ≤10 respondents. In addition to that, slightly >13% of respondents held an additional job. Measures of central tendency and dispersion and variables socio-demographic are shown in Table 1. The detailed information on the measures of central tendency and dispersion in scales AWS, WEIMS-PL and MBI are presented in Table 2. The correlation between net monthly salary and scores on individual scales are shown in Table 3.

**Table 1.** Measures of central tendency and dispersion and variables socio-demographic in the study among 301 nurses, January 2024 – March 2025, Germany

Variable	Min.	Max	M	SD	Q1	Me	Q3	Mode
Age [years]	20.0	70.0	39.6	12.94	30.0	35.0	48.0	30.0
Work experience [years]	1.0	45.0	11.8	10.33	4.0	8.0	17.0	3.0
Net monthly salary [EUR]	2100.0	3900.0	2735.2	302.30	2550.0	2700.0	2900.0	2700.0

Q1 – first quartile; Q3 – third quartile.

**Table 2.** Measures of central tendency and dispersion and scores on individual scales in the study among 301 nurses, January 2024 – March 2025, Germany

Area	Min.	Max	M	SD	Q1	Me	Q3	Mode
*Areas of Worklife Survey* (AWS)								
community	1.6	4.6	3.3	0.62	2.8	3.4	3.8	3.4
values	1.8	4.4	3.3	0.51	3.0	3.4	3.8	3.4
workload	1.5	4.7	3.2	0.73	2.8	3.5	3.8	3.7
control	1.3	4.7	3.2	0.73	2.7	3.3	3.7	3.7
reward	1.3	5.0	3.2	0.83	2.5	3.5	3.8	3.8
fairness	1.3	4.5	3.0	0.68	2.5	3.0	3.5	3.2
*Work Extrinsic and Intrinsic Motivation Scale* (WEIMS-PL)								
integrated regulation	6.0	20.0	13.8	2.63	12.0	14.0	16.0	15.0
identified regulation	5.0	20.0	13.0	3.05	11.0	13.0	15.0	15.0
intrinsic motivation	5.0	18.0	12.9	2.63	11.0	13.0	15.0	14.0
external regulation	4.0	21.0	12.6	3.70	10.0	12.0	15.0	10.0
introjected regulation	4.0	20.0	12.4	3.05	11.0	13.0	15.0	14.0
amotivation	4.0	20.0	11.8	3.61	9.0	11.0	15.0	10.0
*Maslach Burnout Inventory* (MBI)								
personal accomplishment	12.5	95.8	52.5	17.76	37.5	54.2	66.7	58.3
emotional exhaustion	0.0	96.3	36.5	22.11	18.5	33.3	51.9	33.3
depersonalization	0.0	93.3	32.7	18.87	20.0	33.3	40.0	26.7
overall burnout index	7.6	81.8	41.5	13.19	33.3	40.9	50.0	40.9

Q1 – first quartile; Q3 – third quartile.

**Table 3.** Spearman's rank correlation coefficient values: relationship between net monthly salary, age, work experience, overall burnout index and scores on individual scales in the study among 301 nurses, January 2024 – March 2025, Germany

Area	Net monthly salary		Age		Work experience		Overall burnout index (MBI)	
	Spearman's rank correlation coefficient	p	Spearman's rank correlation coefficient	p	Spearman's rank correlation coefficient	p	Spearman's rank correlation coefficient	p
*Areas of Worklife Survey* (AWS)								
workload	–0.03	0.607	–0.57	<0.001	–0.66	<0.001	–0.31	<0.001
control	–0.08	0.148	–0.47	<0.001	–0.64	<0.001	–0.20	<0.001
reward	–0.07	0.231	–0.60	<0.001	–0.75	<0.001	–0.29	<0.001
community	–0.05	0.379	–0.23	<0.001	–0.34	<0.001	–0.10	0.092
fairness	–0.15	0.008	–0.03	0.586	–0.13	0.026	–0.06	0.264
value	–0.08	0.183	–0.29	<0.001	–0.34	<0.001	–0.28	<0.001
*Work Extrinsic and Intrinsic Motivation Scale* (WEIMS-PL)								
amotivation	0.06	0.282	0.52	<0.001	0.66	<0.001	0.14	0.013
external regulation	0.00	0.991	0.53	<0.001	0.63	<0.001	0.19	0.001
introjected regulation	0.05	0.367	–0.47	<0.001	–0.60	<0.001	–0.14	0.014
identified regulation	0.10	0.086	–0.28	<0.001	–0.35	<0.001	–0.08	0.171
integrated regulation	0.04	0.448	–0.28	<0.001	–0.37	<0.001	–0.06	0.310
intrinsic motivation	0.01	0.814	–0.53	<0.001	–0.63	<0.001	–0.20	<0.001
*Maslach Burnout Inventory* (MBI)								
emotional exhaustion	0.08	0.174	0.48	<0.001	0.35	<0.001		
depersonalization	0.06	0.315	0.34	<0.001	0.24	<0.001		
personal accomplishment	0.11	<0.050	–0.15	0.007	–0.14	0.016		
overall burnout index	0.10	0.100	0.39	<0.001	0.28	<0.001		

### Areas of Worklife Survey

In the AWS, respondents achieved the highest mean scores in the community and values scales. On the contrary, the lowest mean scores were noted in the fairness scale. In terms of the community scale in the AWS, the score was M±SD 3.3±0.62 pts. Furthermore, the result obtained for the values scale reached M±SD 3.3±0.51 pts. In the case of the workload scale, the value was calculated at M±SD 3.2±0.73 pts. Regarding the control scale, the score was also M±SD 3.2±0.73 pts. Similarly, the rewards scale showed a score of M±SD 3.2±0.83 pts. The score for the fairness scale was M±SD 3.0±0.68 pts.

### Work Extrinsic and Intrinsic Motivation Scale

As for the obtained answers in WEIMS-PL, the highest mean scores were noted in integrated regulation, identified regulation and intrinsic motivation scales. On the other hand, amotivation scale ranked the lowest.

The integrated regulation scale had an average result of M±SD 13.8±2.63. In the identified regulation scale, the score reached M±SD 13.0±3.05. The intrinsic motivation scale showed a value of M±SD 12.9±2.63. In terms of external regulation, the result was M±SD 12.6±3.70. Finally, the score for the amotivation scale was M±SD 11.8±3.61.

### Maslach Burnout Inventory

Respondents scored the highest on the personal accomplishment scale in the MBI, while the lowest scores were recorded for the depersonalization scale. The overall score for the MBI was M±SD 41.5±13.19 pts.

The personal accomplishment scale showed a score of M±SD 52.5±17.76. In the emotional exhaustion scale, the value amounted to M±SD 36.5±22.11. Finally, the depersonalization scale recorded a result of M±SD 32.7±18.87.

### Statistical analysis

The statistical analysis did not reveal any statistically significant differences in the distribution of scores across individual scales between women and men (p ≥ 0.05).

The correlation analysis (Table 3) revealed statistically significant relationships between age and:

– workload (AWS) – negative correlation (r = −0.57) – younger respondents scored higher, older participants were less likely to believe they could handle their assigned work duties (p < 0.001);
– control (AWS) – negative correlation (r = −0.47) – younger respondents scored higher, older participants were less likely to feel they had autonomy in decision-making (p < 0.001);
– rewards (AWS) – negative correlation (r = −0.60) – younger respondents scored higher, older participants were less satisfied with the rewards they received for their work (p < 0.001);
– community (AWS) – negative correlation (r = −0.23) – younger respondents scored higher, older participants rated their workplace social environment lower (p < 0.001);
– values (AWS) – negative correlation (r = −0.29) – younger respondents scored higher, older participants were less likely to perceive alignment between their values and those promoted by their organization (p < 0.001);
– AMO (WEIMS) – positive correlation (r = 0.52) – older respondents scored higher; it was more probable for them to experience thorough moments of lack of motivation to work (p < 0.001);
– EXT (WEIMS) – positive correlation (r = 0.53) – older respondents obtained higher ranks as compared with the younger ones; the former were more likely to work mostly for external benefits (p < 0.001);
– IDEN (WEIMS) – negative corelation (r = − 0.28) – younger respondents were more likely to perceive work as a means of fulfilling personal values (p < 0.001);
– INTEG (WEIMS) – negative correlation (r = −0.28) – younger respondents scored higher, they were more likely to see work as a key element of their identity (p < 0.001);
– IM (WEIMS) – negative correlation (r = −0.53) – younger respondents scored higher, they exhibited genuine interest in their work (p < 0.001);
– emotional exhaustion (MBI) – positive correlation (r = 0.48) – older respondents scored higher, they experienced emotional exhaustion more frequently (p < 0.001);
– depersonalization (MBI) – positive correlation (r = 0.34) – older respondents scored higher, they were more likely to exhibit indifference towards the problems of others (p < 0.001);
– personal accomplishment (MBI) – negative correlation (r = −0.15) – younger respondents scored higher, it was more common for them to view their personal achievements as lower (p = 0.007);
– overall burnout (MBI) – positive correlation (r = 0.39) – older respondents had higher scores when compared with younger participants of the study; it was more common to experience professional burnout among older participants of the study (p < 0.001).

The correlation analysis (Table 3) revealed the presence of statistically significant relationships between work experience and:

– workload scale (AWS) – negative correlation (r = −0.66) – respondents with shorter work experience scored higher; those with longer experience were less likely to believe they could handle their assigned work duties (p < 0.001);
– control scale (AWS) – negative correlation (r = −0.64) – respondents with shorter work experience scored higher; those with longer experience were less likely to feel they had autonomy in decision-making (p < 0.001);
– rewards scale (AWS) – negative correlation (r = −0.75) – respondents with shorter work experience scored higher; those with longer experience were less satisfied with the rewards they received for their work (p < 0.001);
– community scale (AWS) – negative correlation (r = −0.34) – respondents with shorter work experience scored higher; those with longer experience rated their workplace social environment lower (p < 0.001);
– fairness scale (AWS) – negative correlation (r = −0.13) – respondents with shorter work experience scored higher; those with longer experience were less likely to feel they were treated fairly (p = 0.026);
– values scale (AWS) – negative correlation (r = −0.34) – respondents with shorter work experience scored higher; those with longer experience were less likely to perceive alignment between their values and those promoted by their organization (p < 0.001);
– AMO (WEIMS) – positive correlation (r = 0.66) – respondents with longer work experience scored higher, indicating they more frequently experienced a lack of motivation to work (p < 0.001);
– EXT (WEIMS) – positive correlation (r = 0.63) – respondents with longer work experience scored higher, indicating they more often worked primarily for external benefits (p < 0.001);
– INTRO (WEIMS) – negative correlation (r = −0.60) – respondents with shorter work experience scored higher, experiencing internal pressure more frequently (p < 0.001);
– IDEN (WEIMS) – negative correlation (r = −0.35) – respondents with shorter work experience scored higher, perceiving work more often as a way to fulfil personal values (p < 0.001);
– INTEG (WEIMS) – negative correlation (r = −0.37) – respondents with shorter work experience scored higher, more often seeing work as a key element of their identity (p < 0.001);
– IM (WEIMS) – negative correlation (r = −0.63) – respondents with shorter work experience scored higher, demonstrating more authentic interest in their work (p < 0.001);
– emotional exhaustion (MBI) – positive correlation (r = 0.35) – respondents with longer work experience scored higher, reporting emotional exhaustion more frequently (p < 0.001);
– depersonalization (MBI) – positive correlation (r = 0.24) – respondents with longer work experience scored higher, showing greater indifference towards the problems of others (p < 0.001);
– reduced personal accomplishment (MBI) – negative correlation (r = −0.14) – respondents with shorter work experience scored higher, perceiving their personal achievements as lower (p = 0.016);
– overall burnout (MBI) – positive correlation (r = 0.28) – respondents with longer work experience scored higher, indicating they more frequently experienced professional burnout (p < 0.001).

The correlation analysis revealed statistically significant relationships between received salary and:

– fairness (MBI) – negative correlation (r = −0.15) – respondents with lower salaries scored higher; those with higher earnings rated their workplace social environment lower (p = 0.026);
– personal accomplishment (MBI) – positive correlation (r = 0.11) – respondents with higher salaries scored higher, indicating they more often perceived their personal achievements as lower (p < 0.050).

The statistical analysis did not reveal any additional statistically significant correlations (p ≥ 0.05). However, the statistical analysis showed that respondents without additional employment rated their workplace social environment higher than those with additional jobs (p = 0.002). Moreover, respondents without additional employment were also more likely to feel they were treated fairly compared to those with additional jobs (p = 0.013). Finally, no other statistically significant differences were found in the distribution of scores across the remaining scales between the study groups (p ≥ 0.05).

The correlation analysis revealed statistically significant relationships between the overall burnout and:

– workload (WMS) – negative correlation (r = −0.31) – respondents with lower burnout scores achieved higher results; those experiencing higher levels of burnout were less likely to believe they could handle their assigned work duties (p < 0.001);
– control (WMS) – negative correlation (r = −0.20) – respondents with lower burnout scores achieved higher results; those experiencing higher burnout were less likely to feel they had autonomy in decision-making (p < 0.001);
– rewards (WMS) – negative correlation (r = −0.29) – respondents with lower burnout scores achieved higher results; those experiencing higher burnout were less satisfied with the rewards they received for their work (p < 0.001);
– values (WMS) – negative correlation (r = −0.28) – respondents with lower burnout scores achieved higher results; those experiencing higher burnout were less likely to perceive alignment between their values and those promoted by their organization (p < 0.001);
– AMO (WEIMS) – positive correlation (r = 0.14) – respondents experiencing higher burnout more frequently reported a lack of motivation to work (p = 0.013);
– EXT (WEIMS) – positive correlation (r = 0.19) – respondents experiencing higher burnout more often worked primarily for external benefits (p = 0.001);
– INTRO (WEIMS) – negative correlation (r = −0.14) – respondents experiencing higher burnout were less likely to feel internal pressure (p = 0.014);
– INTR (WEIMS) – negative correlation (r = −0.20) – respondents experiencing higher burnout were less likely to show genuine interest in their work (p < 0.001).

The correlation analysis did not reveal any statistically significant relationships between the remaining scales and the overall burnout index (p ≥ 0.05) (Table 3).

## DISCUSSION

The findings of this study correspond to previous research regarding the relationship between motivation and professional burnout among nurses. For instance, Dyrbye et al. [16] reported that one-third of nurses exhibited substantial symptoms of burnout, while half expressed dissatisfaction with their jobs, which stress the widespread nature of the issue and its direct impact on job retention. In a similar manner, Murat et al. [17] found that nurses experienced high levels of stress and burnout. This was mostly applicable to younger nurses with less experience, who reported feelings of inadequacy in their roles. These findings correspond with the results of the following study, which demonstrated that younger nurses exhibited higher intrinsic motivation but also faced significant job-related stressors, which might lead to an increased risk of burnout.

The impact of workplace conditions on burnout was further emphasized by Zhang et al. [18], who identified high levels of emotional exhaustion and depersonalization among nurses (particularly those working long hours in COVID-19 quarantine units). Their study found that prolonged exposure to such high-pressure environments increased burnout, which is similar to this study's finding that workload negatively correlates with motivation and strongly contributes to professional exhaustion. Moreover, Jumanah et al. [19] observed that nearly half of the nurses surveyed had burnout symptoms, with significant correlations between burnout and workplace settings. Although their research did not establish a direct link connection burnout and demographic factors such as age or gender, it stressed the broader conclusion that burnout is prevalent across healthcare settings and influenced by work-related factors.

The role of demographic and workplace variables in shaping burnout levels was also confirmed by Majid et al. [20], who found that work experience and department significantly influenced burnout risk. The results of this study can correspond with the following research, which demonstrated that nurses with longer work experience reported higher emotional exhaustion, lower intrinsic motivation and an increased reliance on external regulation. This conclusion indicated that sustained exposure to demanding work environments contributes to disengagement over time. Furthermore, Soyler et al. [21] demonstrated that burnout negatively affects perceived work performance and is inversely correlated with internal and external motivation. Their findings correspond with the present study's conclusion that keeping high levels of motivation (mostly intrinsic motivation) is of the utmost importance for mitigating the negative effects of burnout and sustaining professional effectiveness.

The obtained research results clearly indicate that age influences not only the level of motivation but also the way individuals cope with everyday professional demands. This may be due to the fact that older individuals, despite their experience, may feel a greater psychological burden associated with chronic work-related stress. On the other hand, younger nurses, who often demonstrate higher intrinsic motivation, tend to experience uncertainty and overload, especially during the early years of their professional careers. These differences may result from varying adaptive mechanisms and individual expectations regarding professional life. The study indicate that professional burnout does not follow a single, defined pattern – its development may depend on the stage of one's career and the available personal resources. Therefore, preventive measures should take into account both age and length of service. It seems essential to create a work environment that supports not only professional development but also psychological well-being at every stage of a nurse's career. Such an approach may contribute to reducing the risk of professional burnout and improving the quality of care provided by nursing staff.

## CONCLUSIONS

This study examined the relationship between motivation and professional burnout among nurses in Germany. The research proved that higher intrinsic motivation correlated with lower burnout, while external regulation and amotivation were linked to greater emotional exhaustion and depersonalization. Findings from the AWS indicated that community and values were the strongest aspects of workplace satisfaction, whereas fairness was the most problematic. Older and more experienced nurses reported higher burnout levels, with declining intrinsic motivation and increasing reliance on external incentives over time. Burnout was also associated with negative perceptions of workload, autonomy and rewards, while salary had only a weak correlation with overall burnout levels.

The results stress the need for workplace strategies that stimulate and enhance intrinsic motivation, ensure fair treatment and improve working conditions to improve nurse well-being and retention.

## References

[1] Niinihuhta M, Häggman-Laitila A. A systematic review of the relationships between nurse leaders' leadership styles and nurses' work-related well-being. Int J Nurs Pract. 2022;28:e13040. 10.1111/ijn.13040. 35102648 PMC9788052

[2] Wang F, Zhao H, Liang J, Li T, Luo Y, Lu S, et al. Magnetron sputtering enabled synthesis of nanostructured materials for electrochemical energy storage. J Mater Chem A. 2020;8:20260–85. 10.1039/D0TA07644A.

[3] Sabitova A, McGranahan R, Altamore F, Jovanovic N, Windle E, Priebe S. Indicators associated with job morale among physicians and dentists in low-income and middle-income countries: A systematic review and meta-analysis. JAMA Netw Open. 2020;3:e1913202. 10.1001/jamanetworkopen.2019.13202. 31922555 PMC6991249

[4] Maslach C, Leiter MP. New insights into burnout and health care: Strategies for improving civility and alleviating burnout. Med Teach. 2017;39:160–3. 10.1080/0142159X.2016.1248918. 27841065

[5] Richemond D, Needham M, Jean K. The effects of nurse burnout on patient experiences. Open J Bus Manag. 2022;10:2805–28. 10.4236/ojbm.2022.105139.

[6] Maslach C, Jackson SE. Burnout in organizational settings. Appl Soc Psychol Annu. 1984;5:133–53.

[7] Jacobs AW, Hill TD, Tope D, O'Brien LK. Employment transitions, child care conflict, and the mental health of low-income urban women with children. Womens Health Issues. 2016;26:366–76. 10.1016/j.whi.2016.05.003. 27342392

[8] Dimunova L, Sovariova Soosov M, Mohnyanszki F. Work-related factors influencing burnout syndrome in nurses. Clin Soc Work Health Interv. 2018;9:25–30. 10.22359/cswhi_9_1_04.

[9] Farivar S, Esmaeelinezhad O. Can configurations of motivation and extraversion attenuate job demands–job burnout bond? Int J Organ Anal. 2021;29:1225–39. 10.1108/IJOA-03-2020-2075.

[10] Trépanier S, Vallerand RJ, Ménard J, Peterson C. Job resources and burnout: Work motivation as a moderator. Stress Health. 2020;36:433–41. 10.1002/smi.2939. 32141191

[11] Bebiroglu N, Bayot M, Brion B, Denis L, Pirsoul T, Roskam I, et al. An instrument to operationalize the balance between risks and resources and predict job burnout. Int J Environ Res Public Health. 2021;18:9416. 10.3390/ijerph18179416. 34502004 PMC8431336

[12] Tamaela EY, Hetharie JA, Huwae VE. Extension and consequences of burnout antecedent model to job satisfaction of college lecturers who concurrently hold structural positions at private-owned university in islands of Ambon, Indonesia. Rjoas. 2018;77:74–85. 10.18551/rjoas.2018-05.10.

[13] Leiter MP, Maslach C. Areas of worklife survey. APA PsycTests. 2012. 10.1037/t06444-000.

[14] Tremblay MA, Blanchard CM, Taylor S, Pelletier LG, Villeneuve M. Work extrinsic and intrinsic motivation scale: Its value for organizational psychology research. Can J Behav Sci. 2009;41:213–26. 10.1037/a0015167.

[15] Maslach C, Jackson SE. Maslach burnout inventory – ES form. APA PsycTests. 1981. 10.1037/t05190-000.

[16] Dyrbye LN, West CP, Johnson PO, Cipriano PF, Beatty DE, Peterson C, et al. Burnout and satisfaction with work–life integration among nurses. J Occup Environ Med. 2019;61:689–98. 10.1097/JOM.0000000000001637. 31348422

[17] Murat M, Köse S, Savaşer S. Determination of stress, depression and burnout levels of front-line nurses during the COVID-19 pandemic. Int J Ment Health Nurs. 2021;30:533–43. 10.1111/inm.12818. 33222350 PMC7753629

[18] Zhang Y, Wang C, Pan W, Zheng J, Gao J, Huang X, et al. Stress, burnout, and coping strategies of frontline nurses during the COVID-19 epidemic in Wuhan and Shanghai, China. Front Psychiatry. 2020;11:565520. 10.3389/fpsyt.2020.565520. 33192686 PMC7649755

[19] Qedair JT, Balubaid R, Almadani R, Ezzi S, Qumosani T, Zahid R, et al. Prevalence and factors associated with burnout among nurses in Jeddah: A single-institution cross-sectional study. BMC Nurs. 2022;21:287. 10.1186/s12912-022-01070-2. 36289486 PMC9607756

[20] Majid N, Mohd Roslan MA, Suryanto S. Factors contributing to burnout among nurses in Malaysia public hospital. Environ Behav Proceed J. 2024;9:313–9. 10.21834/e-bpj.v9i28.5856.

[21] Soyler S, Gokalp Y, Sahin SK, Yigit P. Effect of internal and external motivation and burnout levels of nurses on work performances: Cross-sectional analysis. Int J Bus Educ Sci. 2019;1:63–9. 10.36096/ijbes.v1i1.108.

